# Diabetic Patients Could Do As Well as Non-Diabetic Patients without Inflammation on Peritoneal Dialysis

**DOI:** 10.1371/journal.pone.0080486

**Published:** 2013-11-18

**Authors:** Rong Xu, QingFeng Han, TongYing Zhu, Yeping Ren, JiangHua Chen, HuiPing Zhao, MengHua Chen, Jie Dong, Yue Wang, ChuanMing Hao, Rui Zhang, Xiaohui Zhang, Mei Wang, Na Tian, HaiYan Wang

**Affiliations:** 1 Renal Division, Department of Medicine, Peking University First Hospital, Institute of Nephrology, Peking University, Key Laboratory of Renal Disease, Ministry of Health, Key Laboratory of Renal Disease, Ministry of Education, Beijing, China; 2 Department of Nephrology, Peking University Third Hospital, Beijing, China; 3 Department of Nephrology, Huashan Hospital of Fudan University, Shanghai, China; 4 Department of Nephrology, Second Affiliated Hospital of Harbin Medical University, Heilongjiang, China; 5 Kidney Disease Center, the First Affiliated Hospital, College of Medicine, Zhejiang University, Hangzhou, China; 6 Department of Nephrology, Peking University People’s Hospital, Beijing, China; 7 Department of Nephrology, General Hospital of Ningxia Medical University, Ningxia, China; Cardiff University School of Medicine, United Kingdom

## Abstract

**Background:**

Diabetic patients on peritoneal dialysis (PD) have lower survival and are more likely complicated with inflammation than their non-diabetic counterparts. Here, we explored the interaction effects between diabetes and inflammation on the survival of PD patients.

**Methods:**

Overall, 2,264 incident patients were enrolled from a retrospective cohort study in China. Patients were grouped according to the baseline levels of high-sensitive C-reactive protein (hsCRP, ≤3 mg/L or >3 mg/L) or serum albumin (SA, ≥38 g/L or <38 g/L). Then, several multivariable adjusted stratified Cox regression models were constructed for these groups to explore the predicted role of diabetes on all-cause or cardiovascular death under inflammatory or non-inflammatory conditions.

**Results:**

Diabetics on PD were more likely to have inflammation than non-diabetics on PD, and they presented with elevated hsCRP (52.7% vs. 47.3%, *P* = 0.03) or decreased SA (77.9% vs. 62.7%, *P* < 0.001) levels. After stratification by size of center and controlling for confounding factors, diabetes was found to predict all-cause death in patients with hsCRP >3 mg/L or SA <38 g/L but not in patients with hsCRP ≤3 mg/L or SA ≥38 g/L. Similarly, the presence of diabetes was an indication of cardiovascular death in patients with hsCRP >3 mg/L or SA <38 g/L. However, if further adjusted by baseline cardiovascular disease, the predicted role of diabetes on death related to cardiovascular disease in patients with SA <38 g/L disappeared.

**Conclusion:**

Diabetic patients could do as well as non-diabetic patients without inflammation on peritoneal dialysis. Active strategies should be implemented to improve inflammation status in diabetic patients on PD.

## Introduction

With the rapid growth of the peritoneal dialysis (PD) population worldwide [1], PD outcomes have improved, and this is reflected in the US Renal Data System (USRDS) 2012 Annual Data Report [2] and the Canadian Organ Replacement Register [3]. However, the rate of survival of diabetic PD patients is still much worse than that of their non-diabetic counterparts [4] and the 5-year survival rate of diabetic PD patients is only 32% versus 50% for non-diabetic PD patients from USRDS data, or 30% versus 60% from ERA-EDTA Registry [2,5]. More recently, two studies reported concordant findings, stating that diabetic patients could obtain better outcomes from hemodialysis (HD) than from PD [3]. Taken together, these data raised the question of whether PD is still an appropriate modality for patients with diabetes. 

Before the above question is answered, we need to explore if diabetics, as a whole, or just subgroups of them with specific characteristics have poorer outcomes than non-diabetic PD patients, which is very important for us to choose appropriate dialysis modality for diabetics. Unfortunately, few data has been published on this issue. All studies on the comparison of mortality between diabetic and non-diabetic PD patients consider all diabetic participants as a whole [6,7,8]. Of note, diabetes-associated endothelial dysfunction and inflammation explained approximately 43% of the increase in CV mortality risk conferred by diabetes [9]. Inflammation was such a significant predictor of all-cause death and fetal or non-fetal cardiovascular events for PD patients [10,11,12] Therefore, we hypothesized that the association of diabetic status and poor outcome varied by the degree of inflammtion. We aimed to verify this in a multi-center retrospective PD cohort by determining the interaction effect between diabetes and inflammation on PD outcomes. Inflammation here was indicated by commonly-recognized markers, including high-sensitive C-reactive protein (hsCRP) and serum albumin (SA) [13,14,15]. This study also may contribute to the screening of a high-risk subgroup of the PD population and individually tailor therapeutic strategies for PD patients with diabetes.

## Methods

### Ethics Statement

The ethics committee of Peking University First Hospital, China approved this study. Written consent was given by the patients for their information to be stored in the hospital database and used for research.

### Participants

This was an add-on multi-center retrospective cohort study of the SSOP (Socioeconomic Status and Outcome In Patients On PD) [16]. The details of center enrollment and subject selection were described in our previous report [16]. In brief, seven PD centers with professional PD doctors and nurses and well-developed databases maintained for at least 3 years were enrolled. These centers were located in five different provinces and four geographical regions (north, northeast, northwest, and east) of China. All incident patients began the PD program within 1 month after catheter implantations were enrolled in this study. All patients in this study were given lactate-buffered glucose dialysate with a twin-bag connection system (Baxter Healthcare, Guangzhou, China). Patients were followed up to death, transferring to HD, transplantation, or end of study period (August 1st, 2011).

### Clinical and demographic measures

Data regarding age, gender, primary renal disease, history of cardiovascular disease (CVD), and the presence of DM were collected at the baseline. DM was defined as a previous diagnosis of DM at a tertiary care hospital, or intake of insulin or anti-diabetic medication at the initiation of PD therapy. CVD was recorded if one of the following conditions was present: angina, class III/IV congestive heart failure (New York Heart Association), transient ischemic attack, history of myocardial infarction or cerebrovascular accident, or peripheral arterial disease [17]. Weight and height were taken and recorded when patients came back to PD centre for their first time of follow-up (generally after 2 to 4 weeks of PD therapy). The body-mass index (BMI) is calculated by dividing weight (in kg) by the square of height (in meters). Systolic and diastolic blood pressure (SBP and DBP), and baseline biochemistry data, including hemoglobin, triglyceride (TG), total cholesterol (TCHO), serum calcium, phosphorus, intact parathyroid hormone (iPTH), SA and hsCRP, were calculated as the mean of measurements obtained during the first 3 months. If patients dropped off PD within 3 months, their baseline biochemistry data were calculated as the mean of all measurements obtained during the PD therapy. The SBP and DBP was measured according to the guidelines presented in the Seventh Report of the Joint National Committee on the Prevention, Detection, Evaluation and Treatment of High Blood Pressure [18]. Biochemistry profile was examined using an automatic Hitachi chemistry analyzer and albumin level was determined by the bromcresol green method. For patients with hypoalbuminemia, corrected serum calcium was calculated using the following formula: measured total serum calcium (mmol/L) + 0.02 × (40 - serum albumin [g/L]). Dialysis adequacy and residual renal function (RRF) were measured during the first 6 months. RRF was defined as the mean of residual creatinine clearance and residual urea clearance. Dialysis adequacy was determined from the total Kt/V and total creatinine clearance. The current size of each center was also recorded.

Patients were followed-up until November 1^st^, 2011. The causes for death and cardiovascular events were determined by clinicians based on clinical presentations and examinations for in-hospital cases, or by interviews with family members for at-home cases. The cardiovascular events included electrocardiographically documented angina, myocardial infarction, heart failure, electrocardiographically documented arrhythmia, transient ischemic attacks, strokes, other thrombotic events or peripheral vascular disease [19]. Sudden death was defined as unexpected natural death within 1 hour of symptom onset and without a prior condition that would appear fatal [20]. In all analyses, we censored follow-up at the transfer to HD, loss to follow-up, renal transplantation or the end of the study.

### Statistical analysis

Continuous data were presented as means with standard deviation except for RRF and hsCRP, which were presented as the median (interquartile range [IQR]) because of high skew. Categorical variables were presented as proportions. Relevant characteristics were compared between diabetics and non-diabetics. Patient data were compared using the *t*-test or the analysis of variance F-test for normally distributed continuous variables, and the chi-square test for categorical variables or the Mann–Whitney U test for skewed continuous variables.

The main goal of this study is to determine whether the effect of DM on PD outcome differs depending on the level of inflammation. So, stratified Cox regression models, with center size as the stratified factor [21] and main effects and interaction effects of DM and inflammation as covariables, were constructed first. Then, PD patients were divided into two groups according to their baseline level of inflammation, which were assessed from hsCRP (cutoff point, 3 mg/L) or baseline SA (cutoff point, 38 g/L, referred as an indicator of protein energy wasting [22]), respectively. Several stratified multivariable Cox regression models were constructed separately in subjects with and without inflammtion, with all-cause or cardiovascular death as the outcome, and center size as the stratified factor. Covariables included into these models were the combination of DM, age, gender, and some well-recognized risk factors indicated in former studies by us and others, including BMI, hemoglobin, residual renal function, and CVD history.

We reported the multivariable adjusted hazard ratios (HRs) with 95% confidence interval (CI). All probabilities were two-tailed, and the level of significance was set at 0.05. Statistical analyses were performed using SPSS for Windows software version 13.0 (SPSS Inc., Chicago, IL).

## Results

### Baseline characteristics

Data from 2264 patients were collected. Their demographic details were as follows: mean age, 58.1 ± 15.5 years; BMI, 22.9 ± 3.6 kg/m^2^; hemoglobin, 103.3 ± 19.4 g/L; hsCRP, 3.0 mg/L (IQR of 1.0–7.3 mg/L); and SA, 35.4 ± 5.4 g/L. Of these patients, 49.3% were men, 37.3% were diabetics, and 40% had CVD at the baseline. 

Compared to non-diabetics, diabetics were older (*P* < 0.001) and more likely to be male (*P* = 0.02). They also had higher BMI values and a higher prevalence of CVD (*P* < 0.001 for both). Diabetics were prone to a significantly more severe extent of inflammation, as reflected in their lower SA (34.1 ± 5.2 vs. 36.3 ± 5.3 g/L, *P* < 0.001), higher hsCRP (3.1 vs. 2.8 mg/L, *P* = 0.001), and higher prevalence of abnormal hsCRP (52.7% vs. 47.3%, *P* = 0.03) or SA (77.9% vs. 62.7%, *P* < 0.001). In addition, patients with diabetes had significantly higher SBP and DBP values, higher serum TG and TCHO, higher serum calcium, lower serum phosphorus and iPTH, and a higher RRF and total creatinine clearance rate than non-diabetics (*P* = 0.001~0.01, Table 1).

**Table 1:** Baseline characteristics between diabetic and non-diabetic patients on peritoneal dialysis.

Variables	**Total (n=2264)**	**DM (n=844)**	**non-DM (n=1368)**	***P***
**Age (years, mean ± SD)**	58.1±15.5	63.3±11.5	54.9±16.8	<0.001
**Male (%)**	49.30%	52.60%	47.40%	0.02
**BMI (kg/m^2^, mean ± SD)**	22.9±3.6	23.9±3.5	22.3±3.5	<0.001
**Cardiovascular disease (%)**	40.00%	50.30%	36.20%	<0.001
**Serum albumin (g/L, mean ± SD)**	35.4±5.4	34.1±5.2	36.3±5.3	<0.001
**Serum albumin <38 g/L (%)**	68.80%	77.90%	62.70%	<0.001
**hsCRP (mg/L, median with interquartile)**	3.0(1.0-7.3)	3.1(1.2-8.4)	2.8(1.0-6.1)	0.001
**hsCRP >3 mg/L (%)**	50.10%	52.70%	47.30%	0.03
**Systolic blood pressure (mmHg, mean ± SD)**	137±21	139±19	137±21	0.03
**Diastolic blood pressure (mmHg, mean ± SD)**	80±13	78±12	81±13	<0.001
**Hemoglobin (g/L, mean ± SD)**	103.3±19.4	104.2±18.2	102.7±20.1	0.1
**Triglyceride (mmol/L, mean ± SD)**	1.87±1.19	2.00±1.35	1.79±1.07	<0.001
**Total cholesterol (mmol/L, mean ± SD)**	4.91±1.27	5.06±1.37	4.81±1.18	<0.001
Serum calcium* (mmol/L, mean ± SD)	2.28±0.25	2.29±0.25	2.27±0.25	0.01
**Serum phosphorus (mmol/L, mean ± SD)**	1.56±0.47	1.51±0.44	1.59±0.48	<0.001
**iPTH (pg/ml, median with interquartile)**	172.0(78.0-323.1)	138.1(68.0-244.5)	205.4(86.0-370.9)	<0.001
**Dialysis dose (ml/day, mean ± SD)**	5838±2331	5942±2729	5770±2030	0.16
**RRF (ml/min, median with interquartile)**	4.7(2.4-7.8)	5.9(3.2-8.9)	4.3(2.1-7.3)	<0.001
**Total Kt/V (mean ± SD)**	2.1±0.6	2.1±0.7	2.0±0.6	0.11
**Total Ccr (ml/min/1.73m^2^/week, mean ± SD)**	75.2±37.3	81.6±37.4	71.3±36.7	<0.001

DM: diabetes mellitus; BMI: body mass index; hsCRP: high-sensitive C-reactive protein; iPTH: intact parathyroid hormone; RRF: residual renal function; Ccr: creatinine clearance rate

* for patients with hypoalbuminemia, corrected serum calcium was calculated using the following formula: corrected serum calcium = measured total serum calcium (mmol/L) + 0.02 × (40 - serum albumin [g/L]).

### All-cause death and cardiovascular death

During the median follow-up time of 26.7 (13.6–44.1) months, 613 patients died. CVD death (38.3%) was a major cause in the entire group, followed by infection (24.5%), malignancy, gastrointestinal bleeding and malnutrition. Malignancy contributed more to deaths among non-diabetics (15.6% vs. 7.2%) than among diabetics. The leading four causes of CVD death in both groups were myocardial infarction, congestive heart failure, cerebral bleeding, and cerebral infarction. The distribution of causes for CVD death was not significantly different between diabetics and non-diabetics (*P* = 0.12–0.89, Table 2).

**Table 2:** Detailed causes of death in the study population.

	**Total (n=613)**	**DM (n=291)**	**non-DM (n=307**)	***P***
**Follow-up (months)**	26.7(13.6-44.1)	24.8(12.4-42.8)	27.8(14.3-44.2)	0.02
**Causes of death**				
**Cardiovascular disease**	235(38.3%)	123(42.3%)	109(35.5%)	0.09
*Myocardial infarction*	*54(23%)*	*32(26%)*	*19(17.4%)*	0.12
*Congestive heart failure*	*45(19.1%)*	*21(17.1%)*	*24(22%)*	0.34
*Cerebral bleeding*	*33(14%)*	*15(12.2%)*	*18(16.5%)*	0.35
*Cerebral infarction*	*30(12.8%)*	*16(13%)*	*14(12.8%)*	0.97
*Arrhythmia*	*11(4.7%)*	*4(3.3%)*	*7(6.4%)*	0.26
*Peripheral arterial disease*	*3(1.3%)*	*2(1.6%)*	*1(0.9%)*	0.63
*Sudden death*	*22(9.4%)*	*13(10.6%)*	*9(8.3%)*	0.55
*Undefined causes*	*37(15.7%)*	*20(16.3%)*	*17(15.6%)*	0.89
**Infection**	150(24.5%)	78(26.8%)	66(21.5%)	0.13
**Malignancy**	72(11.7%)	21(7.2%)	48(15.6%)	<0.001
**Gastrointestinal bleeding**	29(4.7%)	10(3.4%)	18(5.9%)	0.16
**Malnutrition**	30(4.9%)	12(4.1%)	18(5.9%)	0.33
**Miscellaneous**	30(4.9%)	13(4.5%)	17(5.5%)	0.55
**Undefined**	67(10.9%)	34(11.7%)	31(10.1%)	0.53

DM: diabetes mellitusDM: diabetes mellitus

### Effect of DM on the survival of patients on peritoneal dialysis with or without inflammation

One-, 2-, and 5-year survival rates were 85%, 78% and 61% in non-diabetics and 75%, 63% and 45% in diabetics, respectively. Stratified Cox regression analysis showed that DM and inflammation (as assessed from hsCRP and SA) were both independent predictors of all-cause or cardiovascular death (table 3). Every 10 mg/L increase of hsCRP was associated with 6.2% or 6.5% higher risk of all-cause or cardiovascular death, and every 1 g/L increase of SA was associated with 11.3% or 11.5% lower risk of all-cause or cardiovascular death. In addition, there was significant interaction effect between DM and inflammation on all-cause or cardiovascular death (table 3).

**Table 3:** Main effect and interaction effect of DM and inflammation (as assessed from hsCRP or SA) on all-cause or cardiovascular death.

	All-cause death		Cardiovascular death	
	adjusted HR (95% CI)	*P*	adjusted HR (95% CI)	*P*
DM (non-DM as reference)	1.639(1.350-1.989)	<0.001	1.984(1.441-2.732)	<0.001
hsCRP (every 10 mg/L increase)	1.062(1.046-1.077)	<0.001	1.065(1.045-1.086)	<0.001
interaction effect of DM and hsCRP	1.005(1.001-1.009)	0.013	1.005(1.000-1.011)	0.05
DM (non-DM as reference)	2.295(1.104-3.489)	0.032	2.608(1.271-3.946)	0.036
SA (every 1 g/L increase)	0.887(0.856-0.919)	<0.001	0.885(0.866-0.904)	<0.001
interaction effect of DM and SA	1.044(1.012-1.077)	0.007	1.047(1.001-1.093)	0.021

DM: diabetes mellitus; hsCRP: high-sensitive C-reactive protein; SA: serum albumin

After stratification by center size and controlling for confounding factors including age, gender, BMI, hemoglobin, RRF, and CVD history, the presence of DM could predict all-cause death in patients with hsCRP >3 mg/L (adjusted HR, 1.81; 95% CI, 1.34-2.45; *P*<0.001) but not in patients with hsCRP ≤3 mg/L (adjusted HR, 1.35; 95% CI, 0.95-1.91; *P*=0.094). DM also predicted cardiovascular death in patients with hsCRP >3 mg/L (adjusted HR, 1.83; 95% CI, 1.10-3.04; *P*=0.02) but not in patients with hsCRP ≤3 mg/L (adjusted HR, 1.20; 95% CI, 0.68-2.12; *P*=0.524, Table 4).

**Table 4:** Adjusted hazard ratio of risk factors on death in peritoneal dialysis patients divided by hsCRP level.

	hsCRP<=3mg/L		hsCRP>3mg/L	
	Adjusted HR (95% CI)	*P*	Adjusted HR (95% CI)	*P*
***All-cause death***				
Age (every 1 year increase)	1.05(1.03-1.06)	<0.001	1.04(1.03-1.05)	<0.001
Gender (male vs. female)	0.81(0.59-1.13)	0.216	1.16(0.88-1.55)	0.291
BMI (every 1 kg/m^2^ increase)	0.97(0.93-1.02)	0.274	0.97(0.93-1.01)	0.101
Hemoglobin (every 1 g/L increase)	0.98(0.98-0.99)	0.001	0.98(0.97-0.99)	<0.001
RRF (every 1 ml/min increase)	0.992(0.987-0.998)	0.021	0.990(0.985-0.996)	0.001
CVD history (yes vs. no)	2.3(1.62-3.26)	<0.001	1.5(1.11-2.02)	0.008
DM (yes vs. no)	1.35(0.95-1.91)	0.094	1.81(1.34-2.45)	<0.001
***Cardiovascular death***				
Age (every 1 year increase)	1.04(1.02-1.07)	0.001	1.01(1.00-1.03)	0.039
Gender (male vs. female)	0.75(0.44-1.26)	0.276	1.05(0.66-1.65)	0.853
BMI (every 1 kg/m^2^ increase)	1.03(0.96-1.12)	0.398	0.99(0.93-1.06)	0.865
hemoglobin (every 1 g/L increase)	0.99(0.97-1.00)	0.057	0.98(0.97-0.99)	0.027
RRF (every 1 ml/min increase)	0.994(0.974-1.014)	0.055	0.994(0.990-0.999)	0.047
CVD history (yes vs. no)	4.28(2.32-7.89)	<0.001	3.85(2.24-6.61)	<0.001
DM (yes vs. no)	1.2(0.68-2.12)	0.524	1.83(1.10-3.04)	0.02

SA: serum albumin; BMI: body mass index; RRF: residual renal function; DM: diabetes mellitus; CVD: cardiovascular disease

Adjusted HR: adjusted hazard ratio, which was calculated from stratified Cox regression models with center size as the stratified factor

Similarly, DM was an independent predictor of all-cause death in patients with SA <38 g/L but not in patients with SA ≥38 g/L. Regard to cardiovascular death, the estimated HR of DM was noticeably greater than one in patients with SA <38 g/L (*P*=0.088) but this did not meet the pre-defined threshold for significance (table 5). In addition, age, hemoglobin, RRF, and CVD history were also demonstrated to be independent predictors of all-cause or cardiovascular death (table 4 and table 5).

**Table 5:** Adjusted hazard ratio of risk factors on death in peritoneal dialysis patients divided by SA level.

	SA>=38 g/L		SA <38 g/L	
	Adjusted HR (95% CI)	*P*	Adjusted HR (95% CI)	*P*
***All-cause death***				
Age (every 1 year increase)	1.07(1.04-1.09)	<0.001	1.04(1.03-1.05)	<0.001
Gender (male vs. female)	0.66(0.4-1.08)	0.098	1.11(0.89-1.39)	0.360
BMI (every 1 kg/m^2^ increase)	1.01(0.93-1.07)	0.941	0.99(0.93-1.04)	0.611
Hemoglobin (every 1 g/L increase)	0.99(0.98-1.01)	0.084	0.98(0.98-0.99)	<0.001
RRF (every 1 ml/min increase)	0.987(0.976-0.999)	0.041	0.989(0.982-0.996)	0.011
CVD history (yes vs. no)	2.2(1.26-3.84)	0.005	1.64(1.29-2.07)	<0.001
DM (yes vs. no)	1.05(0.59-1.85)	0.873	1.46(1.15-1.84)	0.002
***Cardiovascular death***				
Age (every 1 year increase)	1.04(0.99-1.08)	0.089	1.02(1.01-1.04)	0.002
Gender (male vs. female)	0.80(0.40-1.21)	0.388	1.11(0.77-1.58)	0.581
BMI (every 1 kg/m^2^ increase)	1.09(0.98-1.21)	0.120	0.98(0.93-1.03)	0.452
Hemoglobin (every 1 g/L increase)	0.99(0.98-1.00)	0.048	0.99(0.98-1)	0.008
RRF (every 1 ml/min increase)	0.986(0.975-0.996)	0.041	0.992(0.980-1.004)	0.051
CVD history (yes vs. no)	6.32(2.11-18.89)	0.001	3.06(2.07-4.54)	<0.001
DM (yes vs. no)	0.91(0.33-2.45)	0.851	1.39(0.95-2.04)	0.088

SA: serum albumin; BMI: body mass index; RRF: residual renal function; DM: diabetes mellitus; CVD: cardiovascular disease

Adjusted HR: adjusted hazard ratio, which was calculated from stratified Cox regression models with center size as the stratified factor

## Discussion

DM is gradually becoming the leading cause of end-stage renal disease in developed and undeveloped countries [2,4,5], and investigations into the most appropriate choice of dialysis modality for this population are urgently required. PD therapy has many potential advantages for diabetics, such as the lack of requirement for a vascular access and systemic anticoagulation, better preservation of renal function, and few episodes of hypotension [23]. Despite these advantages of PD for this group, only a small and decreasing proportion of diabetics with high mortality receive PD [24] compared to their non-diabetic counterparts [2,4,5,6,25]. From these large-scale multicenter Chinese patients on PD, we found that the trend of worse outcome for diabetic PD patients was partly due to coexisting inflammation, even after adjustment for potential confounding factors. For those diabetic PD patients without inflammation, DM was not a risk factor for all-cause death or cardiovascular death.

There are several possible explanations for our results. First, the critical role of inflammation might be due to its relationship with cardiovascular lesions [26] and a higher risk for cardiovascular death in patients with chronic kidney disease [10,11]. Our findings were consistent with that of a previous study showing that the survival rate of diabetics did not differ from that of non-diabetics if CVD and protein energy wasting were absent [27]. It is worth noting that the predict role of DM was possibly underestimated to some extent, because CVD history was included in the COX model and that some of the effect upon mortality of DM is likely through this. Second, inflammation was revealed to be closely associated with malnutrition [28], worse glycemic control [29], increased blood pressure [30] and volume overload [31]. All the above factors play great roles in the development and progression of comorbidities such as CVD and infection in patients on PD [32]. Therefore, it is not surprising that inflammation can dramatically amplify the difference in survival rates of diabetic and non-diabetic PD patients. Third, both DM [33] and inflammation [11] have been associated with faster decline of RRF in PD patients, therefore we could not preclude that the rapid loss of RRF in diabetics with inflammation might contribute to a higher all-cause mortality. The same phenomenon was observed in a previous study [34].

Of note, some of the differences between diabetics and non-diabetics are extremely small but statistically significant because of the size of the study (and hence significant power). In addition, although DM had no significant influence on death in non-inflamed patients, the estimated hazards ratio are still noticeably above 1 (adjusted HR for all-cause death of diabetes is 1.35 in non-inflamed patients). This study had enough sample size (over 2,200 patients) and events (totally 613 death but only 7 covariates contained in the Cox model), thus it was less possible that the lack of formal statistical significance was due to lack of power [35]. We speculated that although inflammation could explain a large proportion of effect of diabetes on PD outcome, other factors such as fluid overload, malnutrition, severity of baseline CVD, comorbidities other than DM and CVD, glycemic control status, and so on may also play some role. Constructing a model with all these related factors possibly could help us obtain more in-depth understanding of the association between diabetes and outcome. However, these factors were difficult to be collected in a multicentre retrospective study. 

An interesting finding from this large-scale multicenter PD cohort was the significant influence of inflammation on mortality predictability in diabetic PD patients. Our findings provided valuable information about the underlying reasons for worse outcome in diabetic PD patients, and thus help nephrologists and PD clinicians in the selection of dialysis modality selection. Previous studies [3636,37,38,39] have shown that endothelial dysfunction, insulin resistance, reduced RRF and innate defenses, and even cognitive dysfunction can be improved in diabetic patients via the resolution of inflammation. Therefore, for diabetic PD patients with severe inflammation, interventional strategies are needed in order to improve their CVD outcomes and survival rate.

This study had certain limitations. Firstly, some CVD-related factors, such as oxidative stress, endothelial function, elasticity of peripheral vessels and atherosclerotic plaque could not be examined in this study. In addition, extra blood samples were only stored for participants from Peking university first hospital. Due to budget limitation, more characterized inflammatory marker such as IL-6 had not been measured. Secondly, we should be aware of the possibility of selection bias (only those centres with sufficiently well maintained database were included), vintage bias (duration of DM) and residual confounding factors (severity of baseline CVD, comorbidities other than DM and CVD, glycemic control, etc.) in this retrospective study. Thirdly, only the mean value of measurements obtained during the first 3 months was assessed, the longitudinal change in hsCRP or SA levels was not. However, repeat measurements for inflammatory markers are often impractical, especially in this retrospective multi-center cohort with over 2,000 participants. Forth, as a retrospective observational study, there can be no proof of causality.

In conclusion, our findings support the hypothesis that DM is an independent predictor of mortality in PD patients with greater levels of inflammation. For those without inflammation, diabetic patients could do as well as non-diabetic patients on peritoneal dialysis. Active anti-inflammatory strategies should be employed for PD patients, especially for diabetic PD patients, to improve their outcomes.
