# Correction: Diabetic Patients Could Do As Well as Non-Diabetic Patients without Inflammation on Peritoneal Dialysis

**DOI:** 10.1371/annotation/db652343-0b20-489f-8e7f-d23b13219b88

**Published:** 2013-12-17

**Authors:** Rong Xu, QingFeng Han, TongYing Zhu, Yeping Ren, JiangHua Chen, HuiPing Zhao, MengHua Chen, Jie Dong, Yue Wang, ChuanMing Hao, Rui Zhang, Xiaohui Zhang, Mei Wang, Na Tian, HaiYan Wang

A grant from the Baxter Corporation was incorrectly omitted from the Funding Statement. The Funding Statement should read: "This work is supported by grants from the Baxer Clinical Research Award and Renal Research Grant of Baxter Corp, China, and the ISN Research Award of ISN GO R&P Committee. The funders had no role in study design, data collection and analysis, decision to publish, or preparation of the manuscript." 

